# Adult Stress Promotes Purinergic Signaling to Induce Visceral Pain in Rats with Neonatal Maternal Deprivation

**DOI:** 10.1007/s12264-020-00575-7

**Published:** 2020-09-09

**Authors:** Shufen Hu, Qian Sun, Wan-Jie Du, Jian Song, Xin Li, Ping-An Zhang, Ji-Tian Xu, Guang-Yin Xu

**Affiliations:** 1 Center for Translational Pain Medicine, Institute of Neuroscience, Soochow University, Suzhou, 215123 China; 2 Department of Physiology and Neurobiology, Medical College of Soochow University, Suzhou, 215123 China; 3 Department of Physiology and Neurobiology, College of Basic Medical Sciences, Zhengzhou University, Zhengzhou, 450001 China

**Keywords:** Irritable bowel syndrome, Visceral hypersensitivity, β_2_ adrenergic receptor, P2X3 receptor, Dorsal root ganglion, Stress

## Abstract

Chronic visceral pain is one of the primary symptoms of patients with irritable bowel syndrome (IBS), which affects up to 15% of the population world-wide. The detailed mechanisms of visceral pain remain largely unclear. Our previous studies have shown that neonatal maternal deprivation (NMD) followed by adult multiple stress (AMS) advances the occurrence of visceral pain, likely due to enhanced norepinephrine (NE)-β_2_ adrenergic signaling. This study was designed to explore the roles of P2X3 receptors (P2X3Rs) in the chronic visceral pain induced by combined stress. Here, we showed that P2X3Rs were co-expressed in β_2_ adrenergic receptor (β_2_-AR)-positive dorsal root ganglion neurons and that NE significantly enhanced ATP-induced Ca^2+^ signals. NMD and AMS not only significantly increased the protein expression of P2X3Rs, but also greatly enhanced the ATP-evoked current density, number of action potentials, and intracellular Ca^2+^ concentration of colon-related DRG neurons. Intrathecal injection of the P2X3R inhibitor A317491 greatly attenuated the visceral pain and the ATP-induced Ca^2+^ signals in NMD and AMS rats. Furthermore, the β_2_-AR antagonist butoxamine significantly reversed the expression of P2X3Rs, the ATP-induced current density, and the number of action potentials of DRG neurons. Overall, our data demonstrate that NMD followed by AMS leads to P2X3R activation, which is most likely mediated by upregulation of β_2_ adrenergic signaling in primary sensory neurons, thus contributing to visceral hypersensitivity.

## Introduction

Irritable bowel syndrome (IBS) is a common gastrointestinal disorder characterized by intestinal dyskinesia and associated with chronic abdominal pain [1–3]. The clinical treatment of chronic abdominal pain is very difficult [4–6]. Research on the disease has been slow due to the lack of suitable animal models. Although previous studies have deepened the understanding of the occurrence and regulation of chronic somatic pain [7–10], the exact pathophysiological mechanism of IBS abdominal pain has not been fully elucidated. Thus, effective treatment strategies for the primary symptoms are still limited [11–13].

More and more evidence has demonstrated that severe adverse environmental factors, especially earlier life stressors may be an inducer that leads to visceral hypersensitivity responses in humans and animals in adulthood [14–16]. Our previous studies have shown that neonatal maternal deprivation (NMD) followed by adult multiple stress (AMS) advances the occurrence of visceral hypersensitivity in rats, partly due to enhanced norepinephrine (NE)-β_2_ adrenergic signaling, while NMD alone does not cause visceral hyperalgesia at the age of 6 weeks[17]. The adrenergic signaling pathway plays an important role in regulating stress responses in many organs including the nervous system [18–20]. It has been reported that the activation of adrenergic receptors regulates the transmission of nociceptive information by inhibiting neurons and neurotransmitter release [15]. However, the mechanism by which NE-β_2_ adrenergic signaling induces visceral pain remains unknown.

Adenosine triphosphate (ATP) is an important neurotransmitter in the peripheral and central nervous systems. Its receptors are classified into two subtypes according to the signal transduction mechanisms and molecular structure: the ion channel type P2X receptors (P2XRs) and the metabolic type P2YRs. Recent studies have shown that ATP and its P2XRs may play an important role in nociceptive signaling and pain regulation [21–24]. The P2XR is a ligand-gated non-selective cation channel receptor with seven subtypes, among which P2X3Rs are selectively expressed in primary afferent sensory neurons, such as the trigeminal ganglion and dorsal root ganglion (DRG). They are widely involved in the occurrence and development of inflammatory pain, neuropathic pain, and cancer pain [25–27]. We have previously shown that inflammation not only alters the voltage-dependence of P2XRs and increases ATP currents, but also up-regulates the expression of P2X2Rs and P2X3Rs [28]. In addition, the expression of P2X3Rs in the brain increases significantly in a visceral hyperalgesia model [29]. However, whether P2X3Rs in colon-related DRGs mediate NE-β_2_ adrenergic signaling in visceral hyperalgesia induced by NMD and AMS has not been reported.

Therefore, we hypothesize that NMD followed by AMS enhances NE-β_2_ adrenergic signaling, which activates P2X3R expression and function, thus contributing to visceral hyperalgesia. To test this hypothesis, we investigated the roles of P2X3Rs in colon-related DRGs using the previously-developed animal model [30]. This and future studies might shed light on the clinical mechanisms of visceral pain in patients with IBS.

## Materials and Methods

### Animals

Adult male Sprague-Dawley rats weighing 150–250 g at the age of 6–7 weeks were selected for the present study. Care and handling of these animals were approved by the Institutional Animal Care and Use Committee of the Soochow University and were strictly in accordance with the guidelines of the International Association for the Study of Pain.

### Establishment of the Combined Stress Rat Model

NMD rats and age-matched control rats experienced AMS at the age of 6 weeks. The AMS protocol included cold-restraint stress for 45 min, forced swimming stress for 20 min, and water-avoidance stress for 60 min. Rats rested for 1 h between each stressor. These animals were divided into two groups: Control (CON)+AMS and NMD+AMS. The specific methods to establish the NMD+AMS model are described in our previously-published report [30].

### Measurement of the Visceral Hyperalgesia Response

Chronic visceral hyperalgesia was measured by recording the minimal distention to evoke an abdominal visceromotor response to colorectal distention (CRD) based on previous studies [12]. All behavioral tests were performed in a blinded manner.

### Drug Administration

A317491 (a P2X3R antagonist, 30 nmol/L intrathecally) dissolved in 0.9% normal saline (NS) was directly injected into the NMD+AMS rats once for behavioral experiments and once daily for 7 consecutive days for molecular expression and electrophysiological experiments. Butoxamine (BUTO), a β_2_ adrenergic receptor (AR) antagonist, at 5 mg/kg body weight was intraperitoneally injected into the NMD+AMS rats once daily for 7 consecutive days for molecular expression and electrophysiological experiments. In the electrophysiological experiments, ATP (20 μmol/L) or NE (20 μmol/L) was dissolved in extracellular fluid and directly incubated for 5 min with the acutely-dissociated DRG neurons from CON+AMS rats.

### Western Blotting

DRGs (T13–L2) from AMS-treated control or NMD rats were dissected out to assess the expression of P2X1/2/3Rs and β_2_-ARs. The antibodies used were anti-GAPDH (1:2000, Goodhere, Hangzhou, China), anti-P2X1/2/3R (1:1000, Alomone Labs, Hadassah Ein Kerem, Israel) and anti-β_2_-AR (1:500, Santa Cruz Biotechnology, Texas, USA). Band density was measured using ImageJ software. The P2X1/2/3R and β_2_-AR expression was normalized to GAPDH.

### Real-Time qPCR

Total RNAs were extracted from DRGs (T13–L2) from AMS-treated control or NMD rats with TRIzol (Thermo, Waltham, MA USA). cDNA was synthesized from total RNA using reverse transcription kits (TransGen Biotech, Beijing, China) as directed by the supplier’s instructions. The sequences of the primer pairs for *p2x3r* were: (F) 5′-TTGGGATCATCAACCGAGCC-3′ and (R) 5′-ATGACAAAGACAGAGGTGCCC-3′. The sequences of the primer pairs for *gapdh* were: (F) 5′-TGGAGTCTACTGGCGTCTT-3′ and (R) 5′-TGTCATATTTCTCGTGGTTCA-3′. The control reaction was performed without a DNA template.

### Immunofluorescence Studies

Fourteen micrometer (14 μm) frozen sections of DRGs (T13–L2) were used in the immunofluorescence study as described previously [31, 32]. The primary antibodies were anti-P2X3R (1:100, Novus Biologicals, Minneapolis, USA), anti-β_2_-AR (1:50, Santa Cruz Biotechnology, Texas, USA), gliocyte marker glutamine synthase (1:200, Abcam, Cambridge, UK), neuron marker NeuN (1:50, MAB377, Merck Millipore, Munich, Germany), large neuron marker NF200 (1:200, Abcam), small and medium peptidergic neuron marker CGRP (1:200, Abcam), small and medium non-peptidergic neuron marker IB4^+^ (1:500, Sigma, Munich, Germany). The secondary antibodies were Alexa Fluor 488 (1:100, Life Technologies Inc., Waltham, MA USA) and 555 (1:500, Life Technologies Inc.). Negative controls were without the primary antibodies.

### Acute Dissociation of DRG Neurons and Whole-Cell Patch Clamp Recording

NMD and control rats exposed to AMS were sacrificed by decapitation (~6 weeks). Detailed procedures for the acute dissociation of DRG neurons and patch clamp recording were as described previously [25, 31]. The dissecting solution (in mmol/L) was: 130 NaCl, 5 KCl, 2 KH_2_PO_4_, 1.5 CaCl_2_, 6 MgSO_4_, 10 glucose, 10 HEPES, pH 7.2, osmotic pressure 305 mOsm. The patch-clamp recording normal external solution (in mmol/L) was: 130 NaCl, 5 KCl, 2 KH_2_PO_4_, 2.5 CaCl_2_, 1 MgCl_2_, 10 HEPES, 10 glucose, pH 7.2, osmotic pressure 295–300 mOsm. The pipette solution (in mmol/L) was: 140 K-gluconate, 10 NaCl, 10 HEPES, 10 glucose, 5 EGTA, 1 CaCl_2_, pH 7.25, 292 mOsm. The enzyme solution contained collagenase D (1.8–2.0 mg/mL; Roche, Auckland, Switzerland) and trypsin (1.2–1.5 mg/mL; Amresco, Radnor, PA USA). The membrane properties were recorded by an HEKA EPC10 patch clamp amplifier (HEKA Electronic GmBH; Lambrecht, Germany). Data were recorded and analyzed using FitMaster (HEKA Electronic GmBH).

### Calcium Imaging

DRG (T13–L2) neurons were loaded with fura-2 acetoxymethyl ester (2 μmol/L, Invitrogen, Waltham, MA USA) with F-127 for 90 min at 37°C. The ratio (R) of fluorescence signal measured at 340 nm, divided by the signal at 380 nm, is proportional to the intracellular Ca^2+^ mobilization as described previously [33]. The amplitudes of Ca^2+^ peak responses were computed as the difference between the peak value and the baseline value.

### Data Analysis

Origin 8 software (OriginLab, Northampton, MA) was used for statistical analysis. All data are shown as the mean ± SEM, and the error bars represent the SEM. All data were tested for a normal distribution before analysis. Different data were analyzed by the two-sample *t* test, the Mann-Whitney test, and other statistical analysis methods as appropriate. *P* < 0.05 was considered statistically significant.

## Results

### P2X3Rs are Co-expressed with β_2_-AR in DRG Neurons

**Fig. 1:**
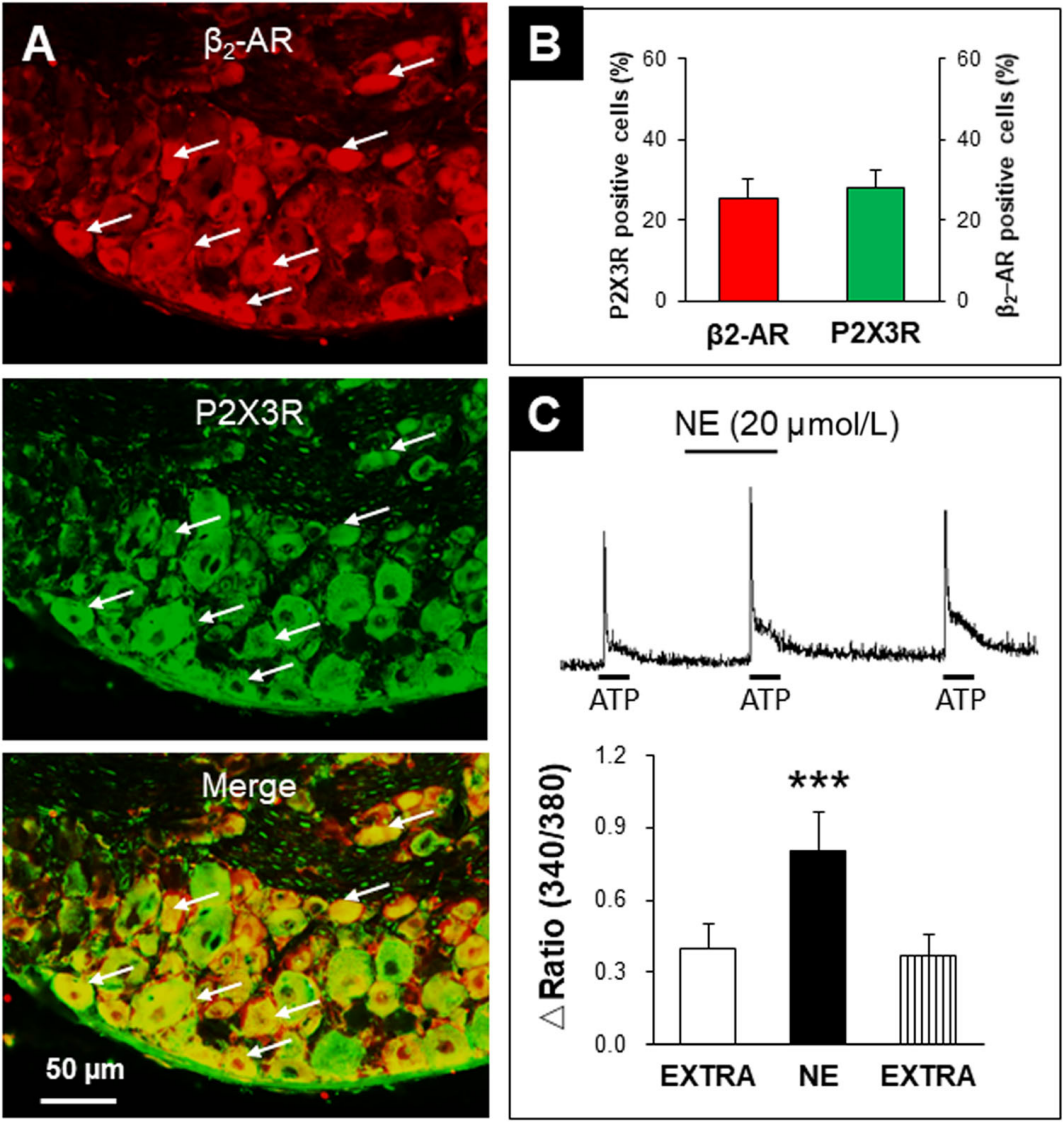
Co-expression of β_2_-ARs with P2X3Rs in DRG Neurons and Enhancement of ATP-induced Ca^2+^ Signaling by NE incubation. **A** β_2_-ARs and P2X3Rs are co-expressed in colorectal-related DRG neurons (red, β_2_-AR-positive cells; green, P2X3R-positive cells; arrows, co-labeled cells; scale bar, 50 μm). **B** Analysis showing that β_2_-ARs are co-expressed with P2X3R-positive DRG neurons, and P2X3Rs are also co-expressed with β_2_-AR-positive DRG neurons (*n =* 3). **C** An example from a cell showing responses to ATP (20 μmol/L) before and after incubation with norepinephrine (NE, 20 μmol/L) for 5 min. NE significantly enhanced the ATP-induced Ca^2+^ signals compared with extracellular solution (EXTRA) (***P < 0.001; *n =* 33, paired *t* test).

Our previous studies have shown that NMD followed by AMS induces visceral hypersensitivity in rats, partly due to enhanced NE-β_2_ adrenergic signaling [30]. So we first determined whether P2X3Rs were co-expressed with β_2_-AR in DRG neurons in CON+AMS rats, and immunofluorescence experiments showed that they were (Fig. 1A). The analysis showed that the percentage of P2X3R-positive cells among all β_2_-AR-positive cells was 25.18%, and the percentage of β_2_-AR-positive cells among all P2X3R-positive cells was 28.03% (Fig. 1B). These results laid a foundation for verifying the interaction between β_2_-ARs and P2X3Rs.

### NE Enhances Calcium Signaling Induced by ATP

Next, the Ca^2+^-imaging technique was used to determine whether NE enhanced P2X3R function. The Ca^2+^ signals induced by ATP (20 μmol/L) significantly increased after incubation with NE (20 μmol/L) in CON+AMS rats (Fig. 1C). The ΔR/R was 0.39 ± 0.11 (*n* = 33) before NE application. Five minutes after NE application, the ΔR/R was 0.81 ± 0.16 (*n* = 33). The ATP-induced intracellular Ca^2+^ mobilization returned to baseline after removal of NE. These data indicated the involvement of adrenergic signaling in the sensitization of P2X3R function.

### P2X3Rs are Predominantly Expressed in DRG Neurons

**Fig. 2:**
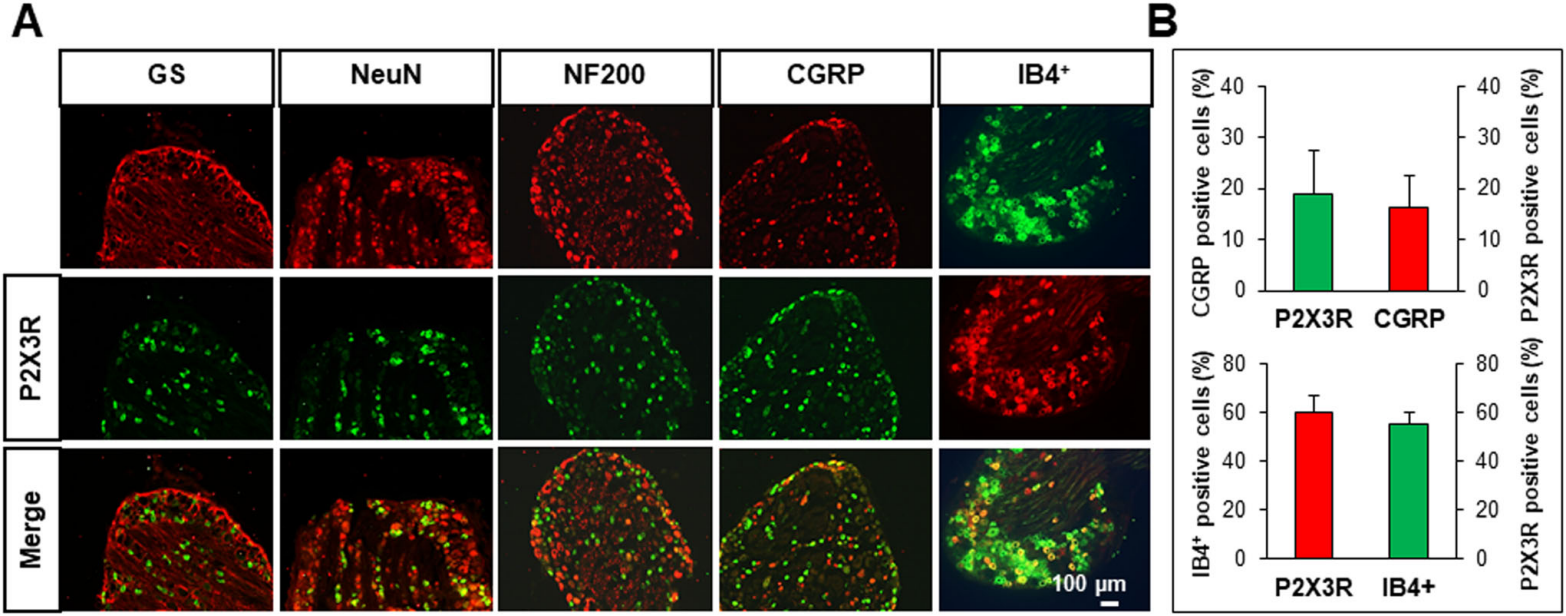
Cellular localization of P2X3R in colorectal-related DRGs in CON+AMS rats. **A** Immunofluorescence results showing that P2X3R has almost no co-labeling with glutamine synthase (GS)-labeled satellite glial cells, but is co-localized with NeuN-labeled neurons. Further P2X3Rs are mainly co-labeled with IB4^+^-labeled small and medium-sized non-peptidergic neurons, and little with NF200-labeled large neurons and CGRP-labeled small and medium-sized peptidergic neurons (scale bar, 100 μm). **B** Analysis showing the majority of P2X3Rs were co-expressed with IB4^+^-positive DRG neurons, and a few were co-expressed with CGRP-positive DRG neurons (*n =* 3).

In order to determine the localization of P2X3Rs in colon-related DRGs of CON+AMS rats, the immunofluorescence technique was used to co-label P2X3Rs and markers of different cell types (Fig. 2A). The results showed that P2X3Rs were mainly expressed in neurons labeled with NeuN, but barely expressed in glial cells labeled with glutamine synthase. Further, P2X3Rs were mainly expressed in small and medium-sized non-peptidergic neurons labeled with the plant lectin IB4^+^, but they were less expressed in large neurons labeled with NF200, and small and medium-sized peptidergic neurons labeled with calcitonin gene-related peptide (CGRP). The analysis showed that the percentages of P2X3R-positive cells among CGRP- and IB4^+^-positive cells were 16.26% and 55.17%, respectively. The percentages of CGRP- and IB4^+^-positive cells among all P2X3R-positive cells were 18.85% and 59.86%, respectively (Fig. 2B).

### NMD and AMS Increases the Expression of P2X3Rs

**Fig. 3:**
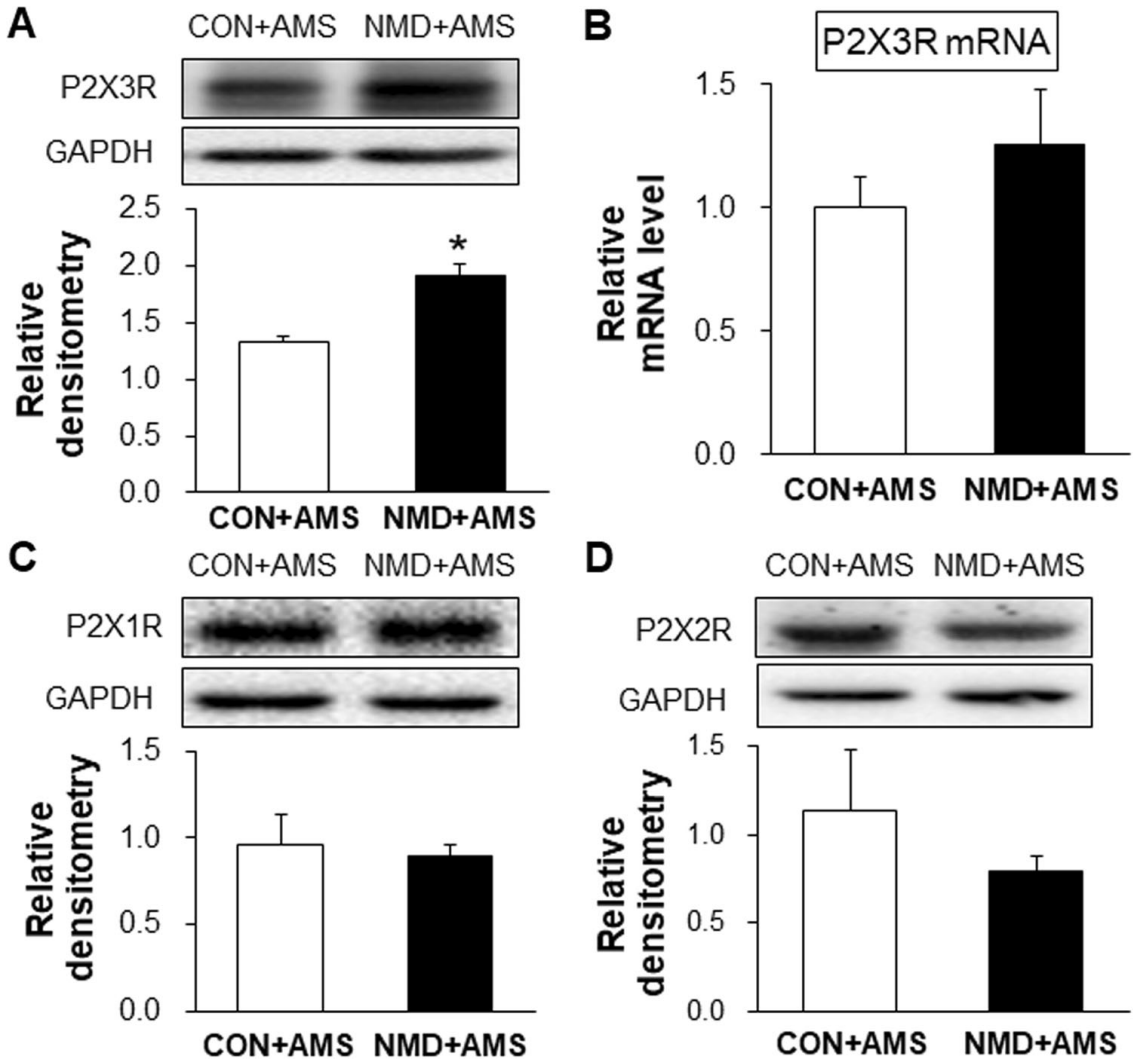
Protein expression of P2X3Rs is significantly increased in NMD+AMS rats. **A** Protein expression of P2X3R is significantly higher in NMD+AMS than in CON+AMS rats (*n =* 3/group; *P < 0.05, two-sample *t* test). **B** There is no change in the mRNA level of P2X3R in NMD+AMS compared to CON+AMS rats (CON+AMS, *n =* 5; NMD+AMS, *n =* 6; *P* > 0.05, two-sample *t* test). **C** There was no change in the protein expression of P2X1R in NMD+AMS compared to CON+AMS rats (CON+AMS, *n =* 4; NMD+AMS, *n =* 3, *P* > 0.05, two-sample *t* test). **D** There is no change in protein expression of P2X2R in NMD+AMS compared to CON+AMS rats (CON+AMS, *n =* 4; NMD+AMS, *n =* 3; *P* > 0.05, two-sample *t* test).

To determine whether P2X3Rs are involved in the formation of chronic visceral pain, we then examined the expression of P2X3Rs in colorectal-related the T13–L2 DRGs of NMD+AMS rats compared with CON+AMS rats. The results showed that the protein expression of P2X3Rs in the NMD+AMS group was significantly higher than that in the CON+AMS group (Fig. 3A). However, the mRNA level was not altered significantly (Fig. 3B). To determine whether the upregulation of P2X3R is specific in this model, we also examined the expression of other purinergic receptors, P2X1Rs and P2X2Rs. The results showed that their protein expression was not altered in the NMD+AMS group compared with the CON+AMS group (Fig. 3C, D). These results suggested that P2X3R expression is specifically enhanced in the T13–L2 DRGs of NMD+AMS rats.

### NMD and AMS Enhance ATP-Induced Currents, the Number of Action Potentials, and Intracellular Calcium Signals

**Fig. 4:**
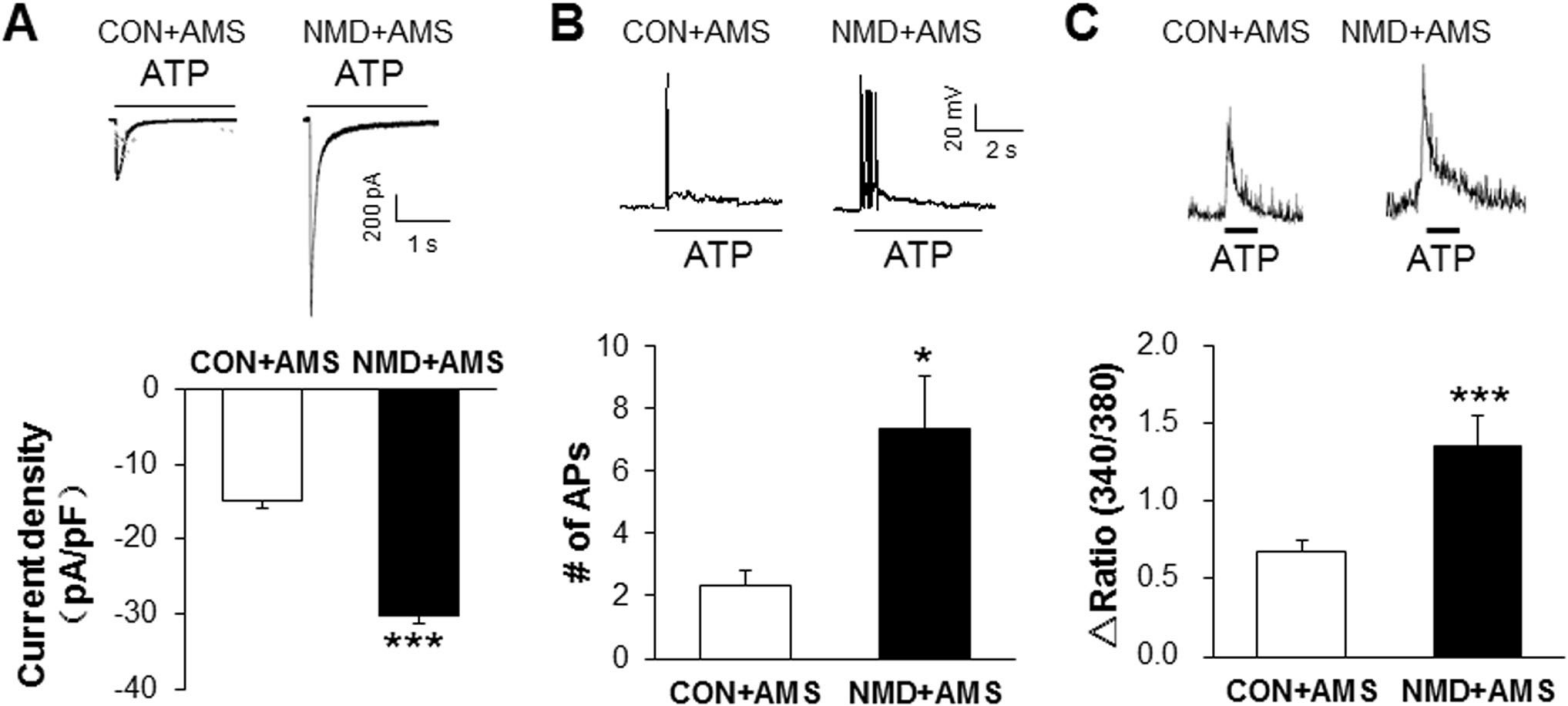
NMD+AMS treatment increases the ATP-induced inward currents, the number of action potentials, and intracellular calcium signals. **A** Typical recordings and statistics of ATP current curves induced by 20 μmol/L ATP stimulation in CON+AMS and NMD+AMS rats. Compared with the CON+AMS rats, the ATP-induced current density is significantly increased in DRG neurons of the NMD+AMS rats (CON+AMS, *n =* 28 cells; NMD+AMS, *n =* 38 cells; ***P < 0.001, Mann-Whitney test). **B** Typical recordings and statistics of action potentials (APs) induced by ATP (20 μmol/L) stimulation in CON+AMS and NMD+AMS rats. Compared with the CON+AMS rats, the ATP-induced APs in the NMD+AMS rats were significantly increased (CON+AMS, *n =* 14 cells; NMD+AMS, *n =* 20 cells; *P < 0.05, Mann-Whitney test). **C** Typical recordings and statistics of Ca^2+^ signals induced by 20 μmol/L ATP stimulation in CON+AMS and NMD+AMS rats. Compared with the CON+AMS rats, the ATP-induced Ca^2+^ signal in the NMD+AMS rats was significantly increased (CON+AMS, *n =* 120 cells; NMD+AMS, *n =* 67 cells; ***P < 0.001, Mann-Whitney test).

Based on the results of high expression of P2X3Rs in NMD+AMS rats, we next determined whether the function of P2X3Rs was changed in the NMD+AMS rats using whole-cell patch clamp recording from small and medium-sized neurons in T13–L2 DRGs. P2X3-mediated currents and action potentials (APs) were induced by application of ATP (20 μmol/L). The results showed that the current density and number of APs induced by ATP in the NMD+AMS group were significantly higher than those in the CON+AMS group (Fig. 4A; CON+AMS, –14.81 ± 2.20 pA/pF; NMD+AMS: –30.33 ± 3.32 pA/pF; Fig. 4B; CON+AMS, 2.36 ± 0.45; NMD+AMS, 7.35 ± 1.71). These results showed that NMD+AMS sensitizes P2X3R function. To confirm this conclusion, ATP-induced the intracellular Ca^2+^ concentration was examined using Ca^2+^ imaging, which showed that the concentration increased significantly after ATP activation in NMD+AMS rats compared to CON+AMS rats (Fig. 4C; CON+AMS, 0.67 ± 0.08; NMD+AMS, 1.35 ± 0.20). These results further demonstrated that P2X3Rs may be involved in the development of visceral hyperalgesia in NMD+AMS rats.

### P2X3R Antagonist A317491 Attenuates Visceral Hyperalgesia and Suppresses the ATP-Induced Calcium Signals

**Fig. 5:**
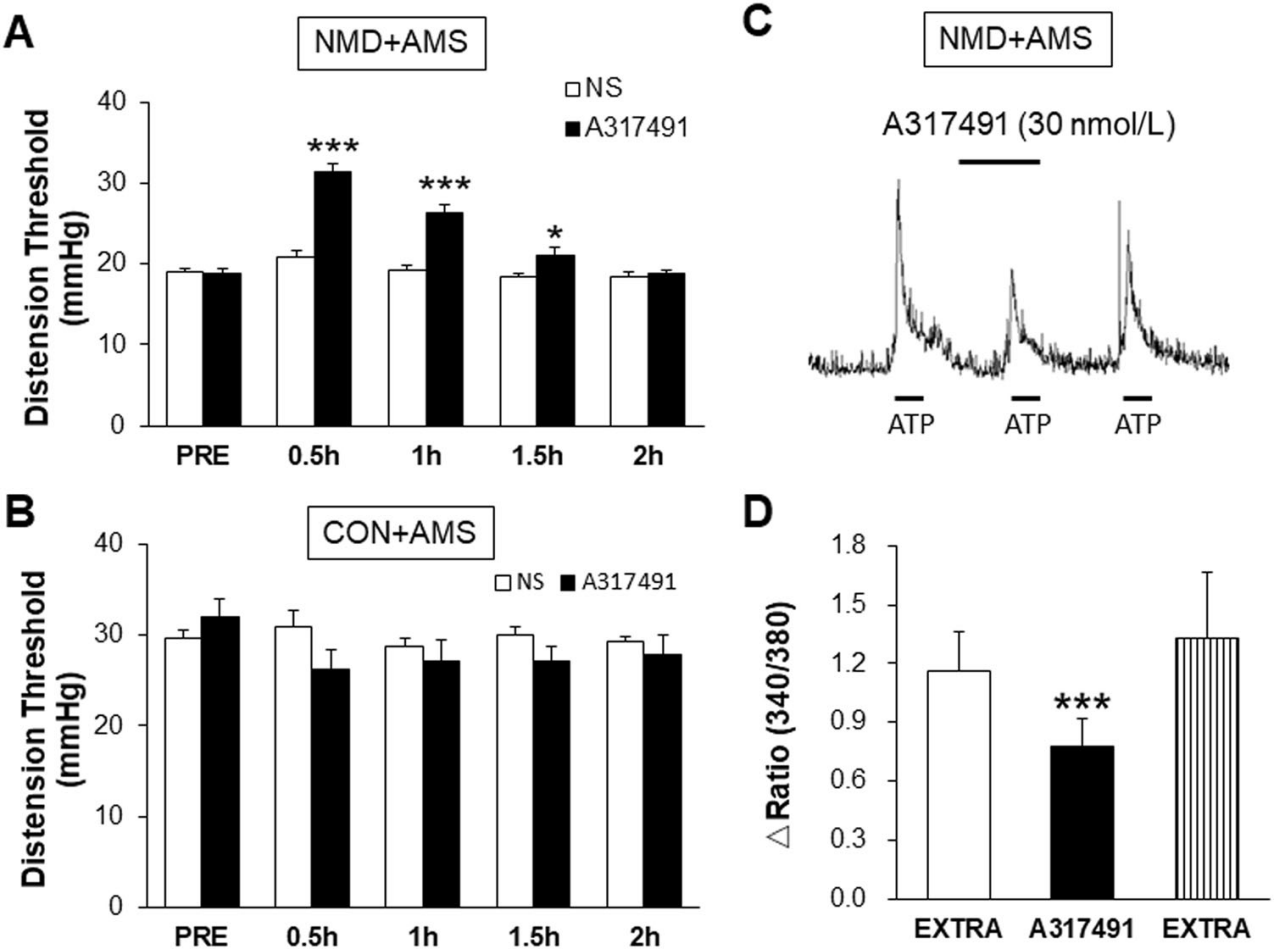
P2X3R antagonist A317491 reverses visceral hyperalgesia and the Ca^2+^ signal in NMD+AMS rats. **A** A317491 significantly increases the CRD threshold of NMD+AMS rats in a time-dependent manner compared with NS (NS, *n =* 10; A317491, *n =* 6; *P <0.05, ***P < 0.001, Tukey’s *post-hoc* test following two -way repeated measures ANOVA). **B** A317491 has no significant effect in CON+AMS rats (NS, *n =* 7; A317491, *n* = 7; *P* > 0.05, Tukey’s *post-hoc* test following two-way repeated measures ANOVA). **C** Example of Ca^2+^ signals from a cell showing responses to ATP (20 μmol/L) before and after incubation with A317491 (30 nmol/L) for 5 min in NMD+AMS rats. **D** Compared with the EXTAR, the ATP-induced Ca^2+^ signal is significantly decreased after incubation with A317491 (*n =* 56 cells/group, ***P < 0.001, paired *t* test).

Our previous study showed that NMD+AMS induces visceral pain [30]. To determine whether P2X3Rs mediate visceral hyperalgesia in NMD+AMS rats, we assessed the analgesic effects of the P2X3R antagonist A317491. The results showed that after intrathecal injection of A317491 (30 nmol/L), the CRD threshold of NMD+AMS rats was significantly increased, and the effect lasted for 1.5 h (Fig. 5A). However, A317491 had no significant effect on CON+AMS rats (Fig. 5B). These results suggest that A317491 specifically acts in NMD+AMS rats and reverses visceral hyperalgesia in a time-dependent manner. Further, we demonstrated that P2X3Rs are involved in the occurrence of visceral pain from a functional point of view. After incubation with A317491 (30 nmol/L), the ATP-induced Ca^2+^ signal was significantly reduced (Fig. 5C, D). These data further suggested that P2X3Rs are involved in pain development.

### β_2_-AR Antagonist Reduces the Expression of P2X3Rs

**Fig. 6:**
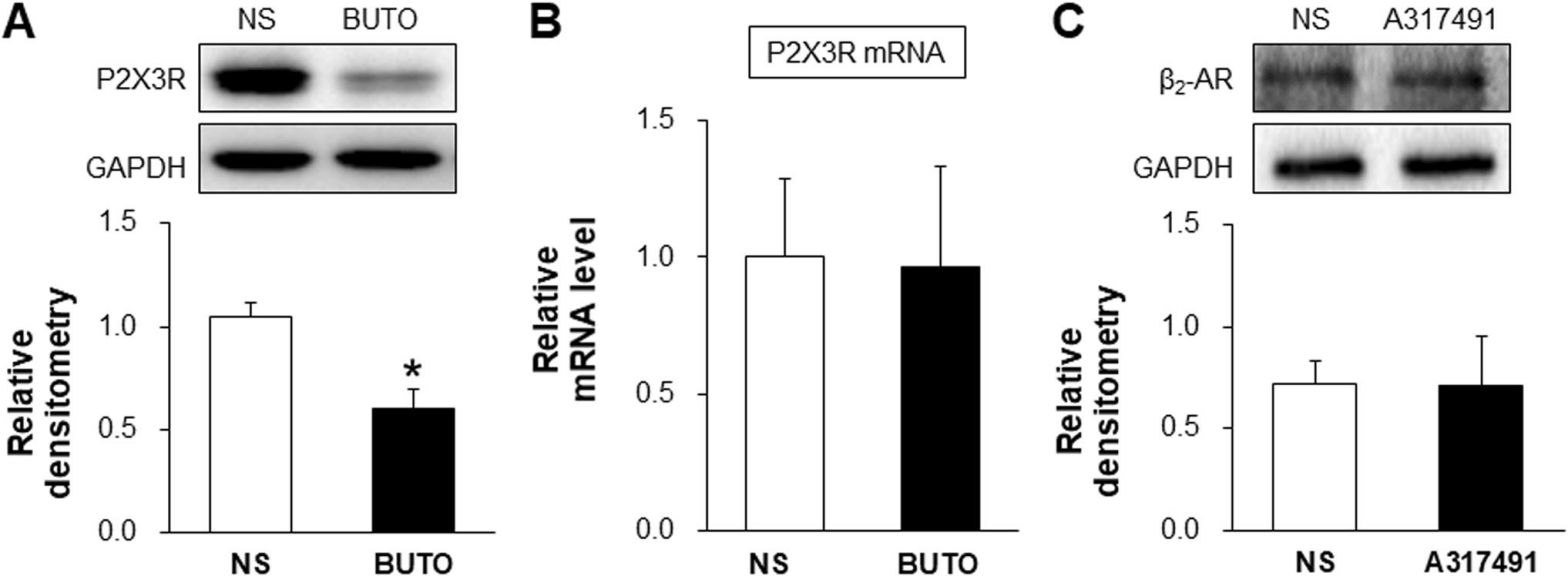
β_2_-AR antagonist decreases the protein expression of P2X3Rs in NMD+AMS rats. **A** BUTO (5 mg/kg) significantly decreases the expression of P2X3R protein in NMD+AMS rats (*n =* 3; *P < 0.05, two-sample *t* test). **B** There was no change in the mRNA level of P2X3R after BUTO injection in NMD+AMS rats (*n =* 3; *P* > 0.05, two-sample *t* test). **C** There was no change in the protein level of β_2_-AR after injecting A317491 for 7 consecutive days in NMD + AMS rats (*n =* 4; *P* > 0.05, two-sample *t* test).

In order to determine the role of the adrenaline signaling pathway in the regulation of P2X3R expression in the T13–L2 DRGs of NMD+AMS rats, we assessed the expression of P2X3Rs after administering butoxamine (BUTO, a β_2_-AR antagonist, 5 mg/kg intraperitoneally once a day for 7 consecutive days) to NMD+AMS rats. The results showed that the protein expression of P2X3Rs in the BUTO group was markedly lower than that in the NS group of NMD+AMS rats (Fig. 6A), but there was no significant change in the mRNA level (Fig. 6B). To determine whether inhibition of P2X3Rs affects β_2_-AR expression, the P2X3R antagonist A317491 was used. Intrathecal injection of A317491 (30 nmol/L, once a day for 7 consecutive days) did not affect the expression of β_2_-ARs in NMD+AMS rats (Fig. 6C). These results suggest that β_2_-AR acts as an upstream molecule that positively regulates the expression of P2X3R protein in visceral hyperalgesia in NMD+AMS rats.

### BUTO Suppresses ATP-Induced Currents and the Number of APs

**Fig. 7:**
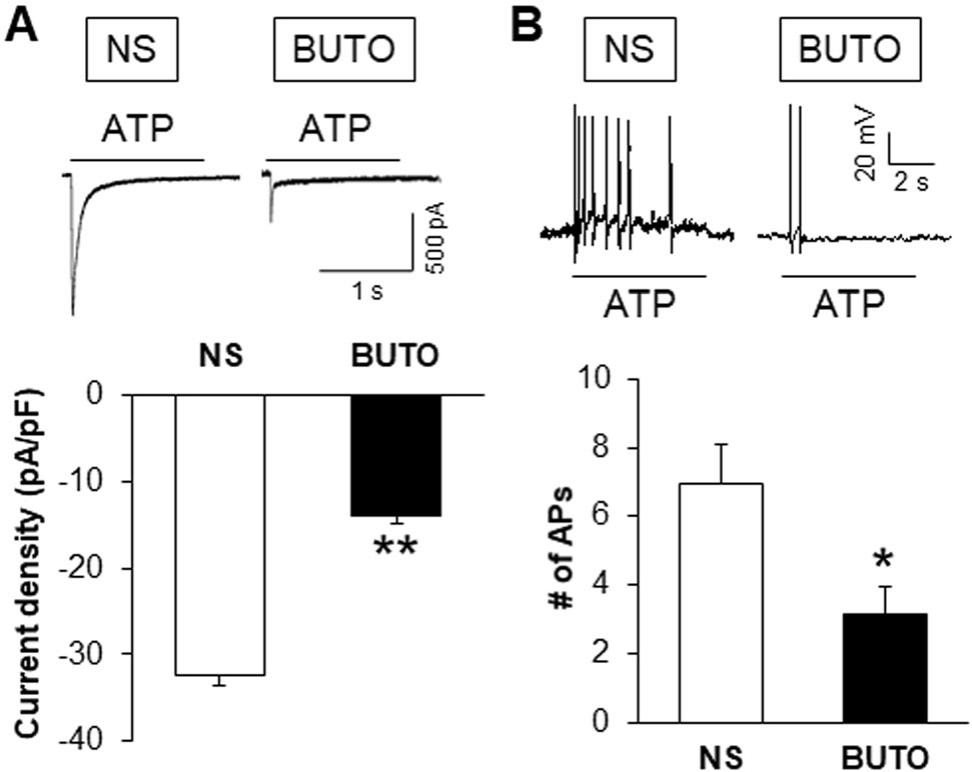
β_2_-AR antagonist reduces ATP-induced current density and intracellular calcium signals. **A** Typical recordings and statistics of the ATP-induced inward currents in NS- and BUTO-treated rats. Injection of BUTO (5 mg/kg) once a day for 7 consecutive days significantly reduced the ATP (20 μmol/L)-induced current density in NMD+AMS rats (NS, *n =* 16 cells; BUTO, *n =* 23 cells; **P < 0.01, Mann-Whitney test). **B** Typical recordings and statistics of APs induced by 20 μmol/L ATP in NS and BUTO rats. Injection of BUTO once a day for 7 consecutive days significantly reduced the number of ATP-induced APs in NMD+AMS rats (NS, *n* = 20 cells; BUTO, *n* = 13 cells; *P < 0.05, Mann-Whitney test).

Last but not least, we explored whether BUTO affects P2X3R activity. The changes of ATP currents and APs were assessed after intraperitoneal injection of the β_2_-AR antagonist BUTO into NMD+AMS rats for seven consecutive days. The results showed that BUTO markedly reduced the ATP-induced current density (Fig. 7A; NS, –32.45 ± 5.70 pA/pF; BUTO, –13.79 ± 1.92 pA/pF) and the number of APs (Fig. 7B; NS, 6.95 ± 1.15; BUTO, 3.15 ± 0.80). These results further demonstrate that β_2_-AR positively regulates the function of P2X3Rs in DRG neurons.

## Discussion

Our previous study confirmed that NMD combined with AMS induced visceral hyperalgesia in rats at 6 weeks and demonstrated that NE signaling is an important mechanism of combined stress-induced visceral hyperalgesia by regulating β_2_-ARs [30]. In the present study, we further confirmed that activation of purinergic P2X3 receptors participates in visceral pain. Importantly, this study explored the possible interaction between adrenergic signaling and purinergic receptors and revealed a new mechanism by which the adrenergic signaling pathway advances the visceral pain, which is most likely mediated by activation of purinergic P2X3 receptors under combined stress conditions.

As a member of the P2X receptor family, purinergic P2X3 receptors are widely expressed in small and medium-sized sensory neurons that transmit nociceptive information [34, 35]. It is a ligand-gated, non-selective cation channel that is activated by extracellular ATP [25, 36]. When ATP activates the P2X3R, the passage of Na^+^, K^+^, and Ca^2+^ are increased, thus leading to an increase in the intracellular Ca^2+^ concentration and the excitability of neurons. Sensitization of P2X3Rs can induce hyperalgesia. Recent studies have shown that P2X3Rs play an important role in the development and progression of inflammatory pain, cancer pain, and neuropathic pain [37, 38]. Our previous studies first proposed that P2X3Rs in the insular cortex participate in IBS-induced visceral hyperalgesia [29]. This study showed that the protein expression of P2X3Rs in T13–L2 DRGs was significantly up-regulated in the NMD+AMS model. The expression of P2X1Rs and P2X2Rs was not significantly altered, indicating a unique role of P2X3 subtype receptors. Further study showed the potentiation of P2X3R function since we showed that the ATP-induced inward current density, number of APs, and intracellular Ca^2+^ concentration were markedly increased in the T13–L2 DRGs of NMD+AMS rats. It has been confirmed that the T13–L2 and L6–S2 DRGs are colon-specific, and previous studies in our laboratory focused on T13–L2 DRGs to explore the mechanisms of visceral hypersensitivity in NMD+AMS rats. So, we chose T13–L2 to assess changes in the molecular expression and neural excitability in the present study. Administration of the potent P2X3R antagonist A317491 significantly reversed the pain threshold of NMD+AMS rats, which is consistent with previous studies [35, 39]. Because the drug reaches the spinal cord after intrathecal injection, we cannot rule out an effect of A317491 on P2X3R in the spinal cord. Since P2X3Rs are predominantly expressed in DRG neurons and A317491 is a potent inhibitor on P2X3Rs, it is reasonable to use this method to evaluate the effect of P2X3Rs in the DRG.

Next, we investigated the mechanisms by which the expression and function of purinergic P2X3Rs were potentiated in NMD+AMS rats. Based on our previous study showing that NE–β_2_-AR signaling plays a critical role in the combined NMD and AMS model, we proposed that the adrenergic receptor β_2_-AR regulates the expression and function of the P2X3Rs in NMD+AMS rats. To test this hypothesis, we first confirmed the co-expression of β_2_-ARs and P2X3Rs in T13–L2 DRG neurons (of course, it would be good to demonstrate that P2X3Rs and β2-ARs are co-expressed in colon-specific DRG neurons by retrograde labeling from the colon and to investigate the percentages that might differ between naïve and NMD+AMS rats in future experiments). Next, we tested their targeting relationship. After intraperitoneal injection of the β_2_-AR antagonist BUTO into NMD+AMS rats for 7 consecutive days, the protein expression of P2X3Rs was suppressed. This result not only supports our previous finding that BUTO suppresses visceral pain [30], but also indicates that adrenergic signaling might be part of the upstream machinery of P2X3R activation. To test this, we used A317491, which did not affect the expression of β_2_-ARs, indicating that P2X3R activation might not be part of the upstream machinery of adrenergic signaling. Together with other findings that application of BUTO suppressed the ATP-induced inward currents and number of action potentials and that NE significantly enhanced the ATP-induced Ca^2+^ signal in T13–L2 DRGs neurons of CON+AMS rats, it is reasonable to conclude that P2X3R activation is most likely mediated by enhanced adrenergic signaling in the combined stress rat model. This conclusion is consistent with our previous study that adrenergic signaling mediates mechanical hyperalgesia by activating the P2X3Rs of primary sensory neuron in rats with chronic pancreatitis [33]. Of note, the suppression of adrenergic signaling did not affect the mRNA levels of P2X3Rs, suggesting that β_2_-AR signaling might regulate P2X3R protein expression after transcription. Whether there are other mechanisms such as roles of microRNAs involved in this regulation needs to be further explored. It is also important to further investigate the detailed molecular mechanism by which the activation of β_2_-ARs promotes purinergic receptor activity under combined stress conditions. In addition, the rapid effect of NE-induced Ca^2+^ signaling might not be mediated by upregulation of P2X3R expression *in vitro*. The purpose of this experiment was to explore the possible interaction of NE and ATP at the DRG neuronal level. This experiment provides a clue that the NE signaling pathway promotes ATP-induced responses. The detailed mechanism of this phenomenon has not been fully investigated. The possible mechanisms might include but may not be limited to the trafficking of P2X3Rs from the cytosolic compartment to the cell membrane and changes in channel kinetics, including increased open probability.

Together with our previously report [30], our findings support the view that the combined stressors enhance NE–β_2_-AR signaling and then activate P2X3R expression and function, thus advancing visceral hyperalgesia. This study helps to elucidate the pathogenesis of visceral hyperalgesia induced by early and adult combined stress. It also suggests that AR inhibitors or P2X3R inhibitors might be alternative drugs to alleviate abdominal pain in IBS patients.

## References

[1] Canavan C, West J, Card T (2014). Review article: the economic impact of the irritable bowel syndrome. Aliment Pharmacol Ther.

[2] Sayuk GS, Gyawali CP (2015). Irritable bowel syndrome: modern concepts and management options. Am J Med.

[3] Drewes AM, Olesen AE, Farmer AD, Szigethy E, Rebours V, Olesen SS (2020). Gastrointestinal pain. Nat Rev Dis Primers.

[4] Dupont HL (2014). Review article: evidence for the role of gut microbiota in irritable bowel syndrome and its potential influence on therapeutic targets. Aliment Pharmacol Ther.

[5] Ji RR. Recent Progress in Understanding the Mechanisms of Pain and Itch: the Second Special Issue. Neurosci Bull 2018, 34: 1–3. 10.1007/s12264-018-0204-z PMC5799133 29340868

[6] Black CJ, Yuan Y, Selinger CP, Camilleri M, Quigley EMM, Moayyedi P (2020). Efficacy of soluble fibre, antispasmodic drugs, and gut-brain neuromodulators in irritable bowel syndrome: a systematic review and network meta-analysis. Lancet Gastroenterol Hepatol.

[7] Bai L, Wang X, Li Z, Kong C, Zhao Y, Qian JL (2016). Upregulation of chemokine CXCL12 in the dorsal root ganglia and spinal cord contributes to the development and maintenance of neuropathic pain following spared nerve injury in rats. Neurosci Bull.

[8] Berta T, Qadri Y, Tan PH, Ji RR (2017). Targeting dorsal root ganglia and primary sensory neurons for the treatment of chronic pain. Expert Opin Ther Targets.

[9] Chen G, Zhang YQ, Qadri YJ, Serhan CN, Ji RR (2018). Microglia in Pain: Detrimental and Protective Roles in Pathogenesis and Resolution of Pain. Neuron.

[10] Chen O, Donnelly CR, Ji RR (2019). Regulation of pain by neuro-immune interactions between macrophages and nociceptor sensory neurons. Curr Opin Neurobiol.

[11] Drossman DA, Camilleri M, Mayer EA, Whitehead WE (2002). AGA technical review on irritable bowel syndrome. Gastroenterology.

[12] Hu S, Xu W, Miao X, Gao Y, Zhu L, Zhou Y (2013). Sensitization of sodium channels by cystathionine beta-synthetase activation in colon sensory neurons in adult rats with neonatal maternal deprivation. Exp Neurol.

[13] Niu HL, Xiao JY (2020). The efficacy and safety of probiotics in patients with irritable bowel syndrome: Evidence based on 35 randomized controlled trials. Int J Surg.

[14] Berrill JW, Sadlier M, Hood K, Green JT (2014). Mindfulness-based therapy for inflammatory bowel disease patients with functional abdominal symptoms or high perceived stress levels. J Crohns Colitis.

[15] Winston JH, Xu GY, Sarna SK (2010). Adrenergic stimulation mediates visceral hypersensitivity to colorectal distension following heterotypic chronic stress. Gastroenterology.

[16] Zhang C, Rui YY, Zhou YY, Ju Z, Zhang HH, Hu CY (2014). Adrenergic beta2-receptors mediates visceral hypersensitivity induced by heterotypic intermittent stress in rats. PLoS One.

[17] Hu S, Xiao Y, Zhu L, Li L, Hu CY, Jiang X (2013). Neonatal maternal deprivation sensitizes voltage-gated sodium channel currents in colon-specific dorsal root ganglion neurons in rats. Am J Physiol Gastrointest Liver Physiol.

[18] Hieble JP, Bondinell WE, Ruffolo RR, Jr. Alpha- and beta-adrenoceptors: from the gene to the clinic. 1. Molecular biology and adrenoceptor subclassification. J Med Chem 1995, 38: 3415–3444. 10.1021/jm00018a001 7658428

[19] Labanski A, Langhorst J, Engler H, Elsenbruch S (2020). Stress and the brain-gut axis in functional and chronic-inflammatory gastrointestinal diseases: A transdisciplinary challenge. Psychoneuroendocrinology.

[20] Orock A, Louwies T, Yuan T, Greenwood-Van Meerveld B. Environmental enrichment prevents chronic stress-induced brain-gut axis dysfunction through a GR-mediated mechanism in the central nucleus of the amygdala. Neurogastroenterol Motil 2020: e13826. 10.1111/nmo.13826 PMC7906280 32084303

[21] Dalgarno R, Leduc-Pessah H, Pilapil A, Kwok CH, Trang T (2018). Intrathecal delivery of a palmitoylated peptide targeting Y382-384 within the P2X7 receptor alleviates neuropathic pain. Mol Pain.

[22] Li P, Zhang Q, Xiao Z, Yu S, Yan Y, Qin Y (2018). Activation of the P2X7 receptor in midbrain periaqueductal gray participates in the analgesic effect of tramadol in bone cancer pain rats. Mol Pain.

[23] Liu C, Zhang Y, Liu Q, Jiang L, Li M, Wang S (2018). P2X4-receptor participates in EAAT3 regulation via BDNF-TrkB signaling in a model of trigeminal allodynia. Mol Pain.

[24] Zhou J, Zhang X, Zhou Y, Wu B, Tan ZY (2019). Up-regulation of P2X7 receptors contributes to spinal microglial activation and the development of pain induced by BmK-I. Neurosci Bull.

[25] Xu GY, Huang LY (2002). Peripheral inflammation sensitizes P2X receptor-mediated responses in rat dorsal root ganglion neurons. J Neurosci.

[26] Zhang HH, Hu J, Zhou YL, Qin X, Song ZY, Yang PP (2015). Promoted interaction of nuclear factor-kappaB with demethylated pPurinergic P2X3 receptor gene contributes to neuropathic pain in rats with diabetes. Diabetes.

[27] Zhou YL, Jiang GQ, Wei J, Zhang HH, Chen W, Zhu H (2015). Enhanced binding capability of nuclear factor-kappaB with demethylated P2X3 receptor gene contributes to cancer pain in rats. Pain.

[28] Labus J, Gupta A, Gill HK, Posserud I, Mayer M, Raeen H (2013). Randomised clinical trial: symptoms of the irritable bowel syndrome are improved by a psycho-education group intervention. Aliment Pharmacol Ther.

[29] Zhang PA, Zhu HY, Xu QY, Du WJ, Hu S, Xu GY (2018). Sensitization of P2X3 receptors in insular cortex contributes to visceral pain of adult rats with neonatal maternal deprivation. Mol Pain.

[30] Du WJ, Hu S, Li X, Zhang PA, Jiang X, Yu SP (2019). Neonatal maternal deprivation followed by adult stress enhances adrenergic signaling to advance visceral hypersensitivity. Neurosci Bull.

[31] Qi F, Zhou Y, Xiao Y, Tao J, Gu J, Jiang X (2013). Promoter demethylation of cystathionine-beta-synthetase gene contributes to inflammatory pain in rats. Pain.

[32] Zhang HH, Hu J, Zhou YL, Hu S, Wang YM, Chen W (2013). Promoted interaction of nuclear factor-kappaB with demethylated cystathionine-beta-synthetase gene contributes to gastric hypersensitivity in diabetic rats. J Neurosci.

[33] Wang S, Zhu HY, Jin Y, Zhou Y, Hu S, Liu T (2015). Adrenergic signaling mediates mechanical hyperalgesia through activation of P2X3 receptors in primary sensory neurons of rats with chronic pancreatitis. Am J Physiol Gastrointest Liver Physiol.

[34] Tao J, Liu L, Fan Y, Wang M, Li L, Zou L (2019). Role of hesperidin in P2X3 receptor-mediated neuropathic pain in the dorsal root ganglia. Int J Neurosci.

[35] Yi Z, Rao S, Ouyang S, Bai Y, Yang J, Ma Y (2017). A317491 relieved HIV gp120-associated neuropathic pain involved in P2X3 receptor in dorsal root ganglia. Brain Res Bull.

[36] Xu GY, Shenoy M, Winston JH, Mittal S, Pasricha PJ (2008). P2X receptor-mediated visceral hyperalgesia in a rat model of chronic visceral hypersensitivity. Gut.

[37] Jiang Q, Li WX, Sun JR, Zhu TT, Fan J, Yu LH (2017). Inhibitory effect of estrogen receptor beta on P2X3 receptors during inflammation in rats. Purinergic Signal.

[38] Weng ZJ, Wu LY, Zhou CL, Dou CZ, Shi Y, Liu HR (2015). Effect of electroacupuncture on P2X3 receptor regulation in the peripheral and central nervous systems of rats with visceral pain caused by irritable bowel syndrome. Purinergic Signal.

[39] Hansen RR, Nasser A, Falk S, Baldvinsson SB, Ohlsson PH, Bahl JM (2012). Chronic administration of the selective P2X3, P2X2/3 receptor antagonist, A-317491, transiently attenuates cancer-induced bone pain in mice. Eur J Pharmacol.

